# Identification of novel PTP1B inhibitor for the treatment of LPS-induced myocardial apoptosis: machine learning based virtual screening and biological evaluation

**DOI:** 10.1080/14756366.2025.2596950

**Published:** 2025-12-10

**Authors:** Xu Dong, Bing Shang, Xin Li, Jie Zhang, Zhen Liu, Bo Feng

**Affiliations:** ^a^ Department of Pharmacy, The Affiliated Hospital of Yangzhou University, Yangzhou University, Yangzhou, China; ^b^ Department of Pharmacy, National Cancer Center/National Clinical Research Center for Cancer/Cancer Hospital, Chinese Academy of Medical Sciences and Peking Union Medical College, Beijing, China; ^c^ State Key Laboratories of Natural and Biomimetic Drugs and School of Pharmaceutical Sciences, Peking University Health Science Center, Beijing, China; ^d^ Institute of Materia Medica, Chinese Academy of Medical Sciences & Peking Union Medical College, Beijing, China; ^e^ Department of Neurology, The Affiliated Hospital of Yangzhou University, Yangzhou University, Yangzhou, China

**Keywords:** PTP1B inhibitor, sepsis induced cardiovascular dysfunction, virtual screening

## Abstract

Sepsis-induced cardiovascular dysfunction (SICD) poses a serious threat to human life. Protein tyrosine phosphatase 1B (PTP1B) displays an essential role in SICD occurrence, so discovering novel inhibitors targeting PTP1B is an effective strategy for SICD treatment. In this research, we exploited a novel virtual screening pipeline consisting of both ligand-based and structure-based modules to find novel PTP1B inhibitors, and compound PI-2 with IC_50_ = 4.1 ± 0.3 μM was successfully discovered. Enzymatic and cellular thermal shift assay showed PI-2 displayed a moderate PTP1B inhibitory activity and a good selectivity towards both PTP1B and TCPTP. Besides, PI-2 effectively protected Lipopolysaccharide (LPS) induced AC16 injury by reducing cell ROS levels and enhancing mitochondrial membrane potential. Overall, this research not only provides a novel virtual screening strategy for discovering novel PTP1B inhibitors, but also supplies a potential candidate for further optimisation for the treatment of SICD.

## Introduction

Sepsis induced cardiovascular dysfunction (SICD) is one of the major complications in sepsis patients, posing a serious threat to human life^1^. Epidemiological investigations have shown that SICD has become the main indicator of poor prognosis in sepsis patients, with a mortality rate as high as 70%-90%, which is 2–3 times higher than that of ordinary sepsis patients^2^. However, the clinical management of SICD remains highly limited; exploring the molecular mechanisms and promising therapeutic targets, along with potential pharmacological interventions, holds significant values^3^.

SICD primarily manifests as impaired myocardial systolic and diastolic function, and a reduced left ventricular ejection fraction (LVEF%) is the most notable indicator for patients^4^. Consequently, restoration of myocardial contractility is critical for SICD prevention and treatment^5^. Mitochondria are the main source of cardiac energy, which is important for myocardial contraction^6^. Various studies have shown that mitochondrial dysfunction is a key factor for cardiac depression; therefore, improving myocardial mitochondrial function is the central focus of SICD treatment^7–9^.

Protein tyrosine phosphatase 1B (PTP1B), an essential member of protein tyrosine phosphatase (PTP) superfamily^10^^,^^11^, has been commonly recognised as a negative regulator of insulin and leptin receptor signaling^12–15^. Recently, a growing number of evidences has proved that PTP1B is a promising target for mitochondrial functional adjustment^16–18^. Inhibition of PTP1B promotes mitochondrial homeostasis in both diabetic cardiomyopathy and calcific aortic valve disease^19^^,^^20^.

Despite, the regulatory function of PTP1B on mitochondria in SICD being unclarified, the importance of PTP1B in SICD treatment is well defined^21^. Studies have shown that deletion of PTP1B attenuates the activation level of p38 mitogen-activated protein kinase (p38 MAPK) and promotes phosphatidylinositol 3-kinase (PI3K)/protein kinase B (Akt) phosphorylation to alleviate SICD^22^^,^^23^ illustrating that PTP1B is an essential target for SICD treatment.

In the past decade, numerous PTP1B inhibitors, containing carboxylate, sulphonate, and difluorophosphate groups, have been developed^24–28^. These inhibitors display preferable PTP1B inhibitory activities; However, none of these compounds have been approved for market because of lacking PTP1B selectivity and poor druggability induced by the high electrification and homology of the binding pocket of PTP1B with other PTPs. Among them, compound Tegeprotafib and Osunprotafib are the only two PTP1B inhibitors which are in clinical phase I for cancer treatment, both of them are also potent T-cell protein tyrosine phosphatase (TCPTP) inhibitors^29^, and compound Ertiprotafib and Trodusquemine are failed in clinical trials for diabetic treatment because of insufficient therapeutic effect or occurrence of side effects (Table 1) ^30^. Despite, PTP1B has already identified as a potential target for SICD treatment, the studies of the anti-SICD activities and mechanisms of these compounds are limited, so developing novel PTP1B inhibitors and clarifying their activities in mitochondria for SICD is still valuable.

**Table 1.** Reported PTP1B inhibitors that in clinical trial.

Compounds	Inhibitory activity (IC_50_)		Phase	Indication
	PTP1B	TCPTP		
Tegeprotafib	1-10 nM	4.4 nM	I	cancer
Osunprotafib	2.5 nM	1.8 nM	I	cancer
Ertiprotafib	1.6 μM		Failed in Phase II	diabetes
Trodusquemine	1 μM	224 μM	Failed in Phase II	diabetes

To discover novel PTP1B inhibitors and evaluate the anti-SICD activities of the novel compound, a virtual screening pipeline consisting of pharmacophore filtering and molecular docking was established. To improve the screening efficiency, Naïve Bayesian Classification (NBC) was used to achieve the requirements of high enrichment of virtual screening. Then, a series of biological assays were conducted to elucidate the PTP1B inhibitory activity and selectivity on the enzymatic and cellular levels. Eventually, cell apoptosis assay, inflammatory cytokine assay, ROS detection, and mitochondrial membrane potential test were used to evaluate the anti-LPS induced myocardial injury potential and mechanisms of the compound with preferable PTP1B inhibitory activity.

## Results and discussion

### PTP1B crystal structure selection

**Figure 1.**
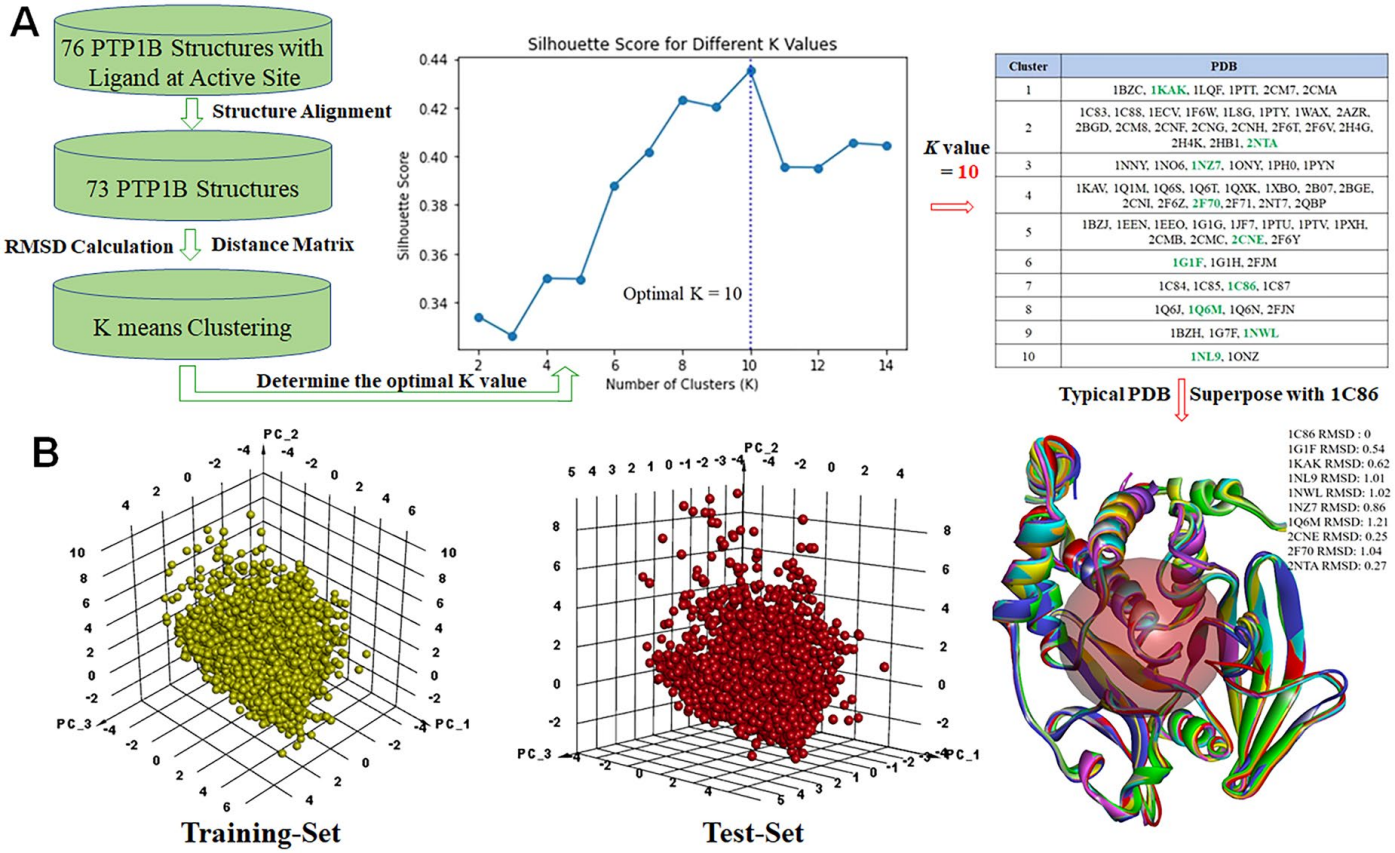
PTP1B crystal cluster and inhibitor database analysis. A. The clustering results of collected PTP1B crystals (The PTP1B was divided into 10 clusters and the representative protein was shown in green); B. Chemical space analysis of the training set (yellow) and the test set (red) by principle component analysis.

Around 367 PTP1B crystal structures (homo sapiens, resolution < 2.5) were listed in RCSB Protein Data Bank (https://www.rcsb.org/). Only 76 PTP1B structures were presented with ligands at the active site. After structure alignment, 73 PTP1B crystals were kept. According to the RMSD and distance matrix, these 73 structures were further clustered by K-means algorithm. As shown in Figure 1(A), when *K* = 10, the silhouette score was maximum, so these crystals were divided into 10 clusters and proteins whose RMSD value was closest to the centroid within the same category was selected. Finally, 1KAK^31^, 2NTA^32^, 1NZ7^33^, 2F70^34^, 2CNE^35^, 1G1F^36^, 1C86^37^, 1Q6M^38^, 1NWL^39^ and 1NL9^40^ were chosen for further study.

### Database diversity analysis

Eventually, a database containing around 26 thousand compounds was generated. The database was further divided into training set and test set in the ratio of 4:1. Generally, the predictive ability of machine learning is affected by the quality of the database, so the principle descriptors including, ALogP, molecular weight, number of hydrogen donors, number of hydrogen acceptors, number of rotatable bonds, number of rings, number of aromatic rings, molecular fractional polar surface area were calculated and analysed. As shown in Figure 1(B), the physical and chemical properties of molecules in both the training set and the test set were distributed uniformly, indicating that the constructed database displayed a good chemical diversity.

### Pharmacophore generation and evaluation

The pharmacophores were generated according to the crystal complex of 1C86, 1G1F, 1KAK, 1NL9, 1NWL, 1NZ7, 1Q6M, 2CNE, 2F70, and 2NTA. The pharmacophores with the highest training set AUC value for each complex were listed in Table 2. We could see that the predictive ability of these pharmacophores was significantly different. Four pharmacophores produced by 1C86, 1NL9, 1NWL, and 1Q6M displayed a good “discriminatory power” with AUC_Training value > 0.7. Among them, 1C86 exhibited the best predictive ability with AUC_Traing value = 0.856, which was much better than 1G1F and 2CNE. To further validate the “discriminatory power” of these pharmacophores, the AUC value of the test set was also calculated. Consistent with the training set, pharmacophores generated by 1C86, 1NL9, 1NWL, and 1Q6M also presented a good “discriminatory power” for the test set.

**Table 2.** Pharmacophores generated by selected PTP1B crystal structures.

PDB Entry	Feature_Set	Selectivity_Score	AUC_Training	AUC_Test
1C86	AANNR	11.689	0.856	0.854
1G1F	ADDNNN	16.495	0.556	0.661
1KAK	AAHHN	9.3394	0.623	0.568
1NL9	AADDHN	13.026	0.724	0.741
1NWL	AAAADH	10.379	0.755	0.789
1NZ7	ADHNNP	15.583	0.549	0.721
1Q6M	AAHHNN	12.590	0.781	0.798
2CNE	AADDNN	14.349	0.591	0.703
2F70	AAADDN	13.301	0.675	0.622
2NTA	AAADHR	10.173	0.660	0.597

A: hydrogen bond acceptor; N: Negative ionisable group; R: Aromatic ring; D: Hydrogen bond donor; H: Hydrophobic group; P: Positive ionisable group; AUC_Training: the AUC value of the training set; AUC_Test: the AUC value of the test set.

**Figure 2.**
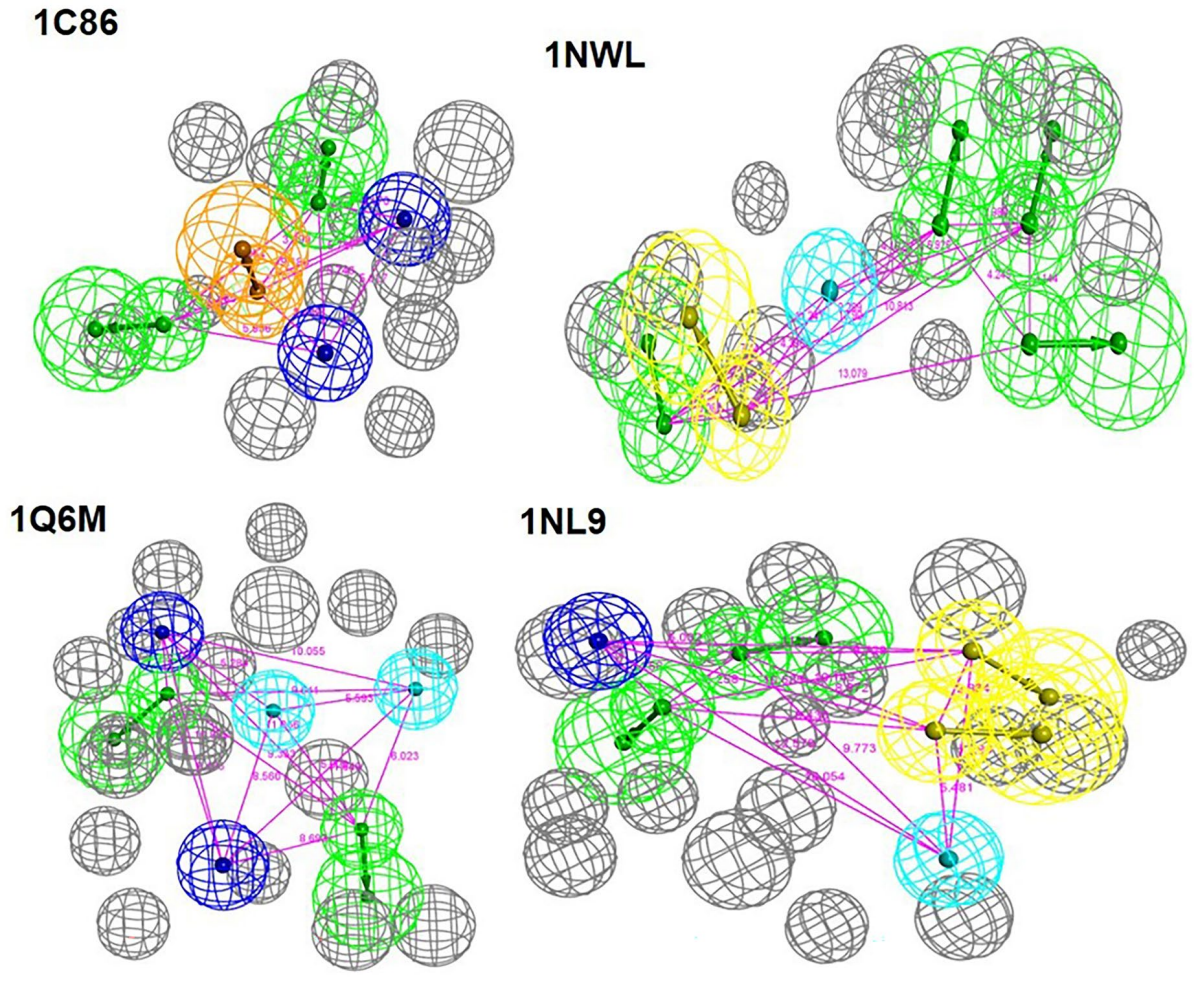
Pharmacophores produced by selected PTP1B crystal (Green: hydrogen bond acceptor; light blue: Hydrophobic group; dark blue: Hydrophobic group; yellow: Hydrogen bond donor; orange: Aromatic ring; gray: steric hindrance).

As shown in Table 2 and Figure 2, the pharmacophore generated by 1C86 consisted of 5 features, including two hydrogen bond acceptors, two negative ionisable groups, and one aromatic ring. The negative ionisable group was also presented in pharmacophores produced by 1Q6M and 1NL9. Despite the negative ionisable feature was beneficial for improving PTP1B inhibitory activity, it was not a preferable fragment for ameliorating membrane permeability, which was one of the main reasons why most reported PTP1B inhibitors displayed poor activity *in vivo*, so the pharmacophore produced by 1NWL, which consisted of four hydrogen bond acceptors, one hydrogen bond donor and one hydrophobic group was chosen for further study.

### Validation of docking reliability

**Figure 3.**
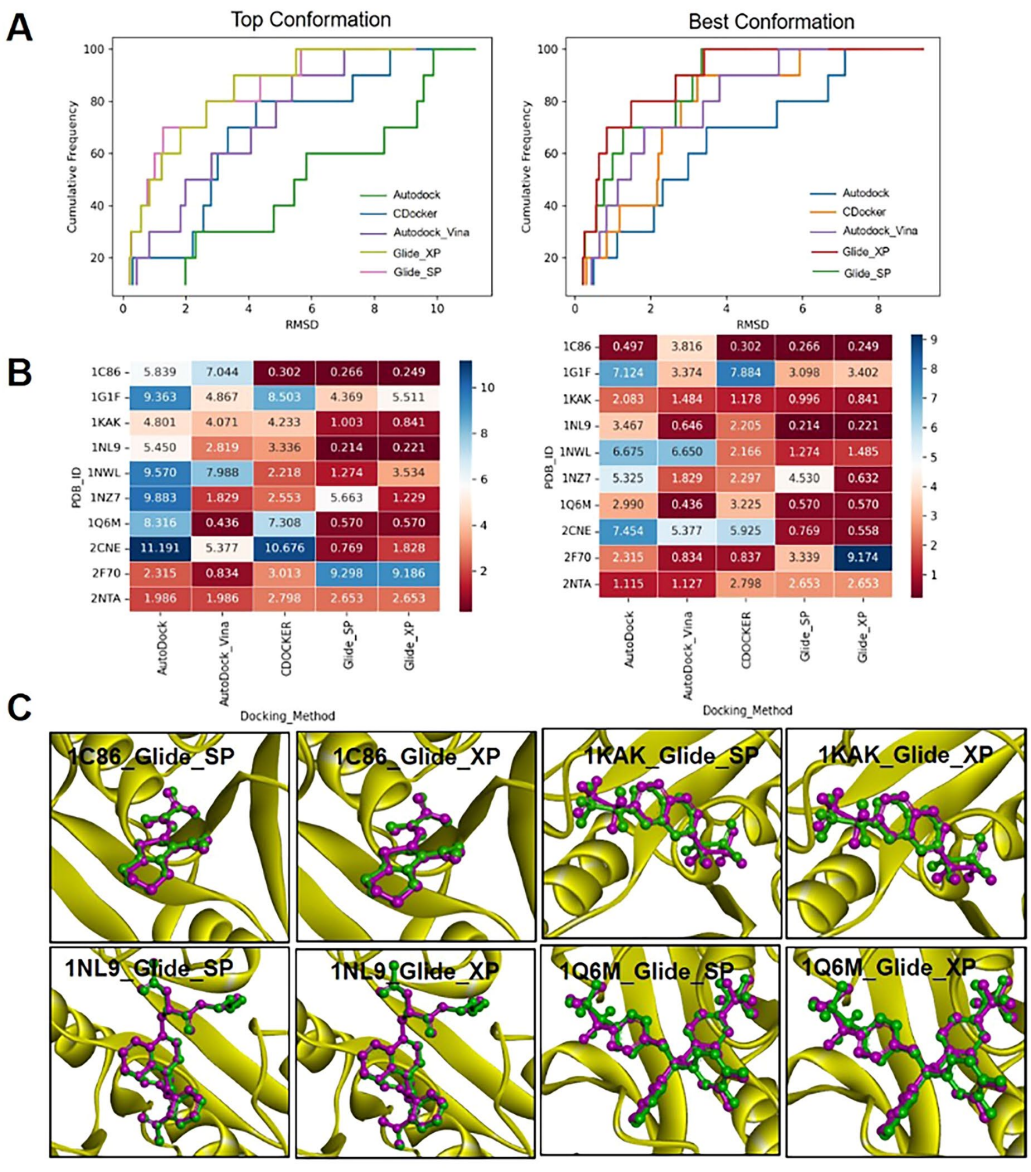
The docking reliability of various docking programs. A. The RMSD cumulative distribution of top-scored poses (Top Conformation) and best poses (Best Conformation) with various docking programs; B. RMSD of top-scored poses (left) and best poses (right) of all docking programs (From red to blue the RMSD increased); C. The superpose of the best poses (green, docked by Glide_SP and Glide_XP) and natural pose (Pink) of each ligand.

The docking reliability was reflected by the ability of docking programs to predict the correct conformation in the molecular docking. The RMSD between the docking pose and the crystal conformation of each ligand was calculated and analysed. As shown in Figure 3(A), Glide_SP and Glide_XP performed superior to other programs in terms of the success rates for the top-scored poses (poses with the highest docking score) and best poses (poses with the lowest RMSD with native conformation). We could see that in Figure 3(B), the top-scored poses were the best poses for 1C86, 1KAK, 1NL9, 1Q6M, and 2NTA. However, the RMSD for 2NTA was > 2 Å, which was considered a failed to predict. Eventually, 1C86, 1KAK, 1NL9, 1Q6M (Figure 3C) were chosen for further study.

### Docking accuracy evaluation and multi-conformational NBC model construction

**Figure 4.**
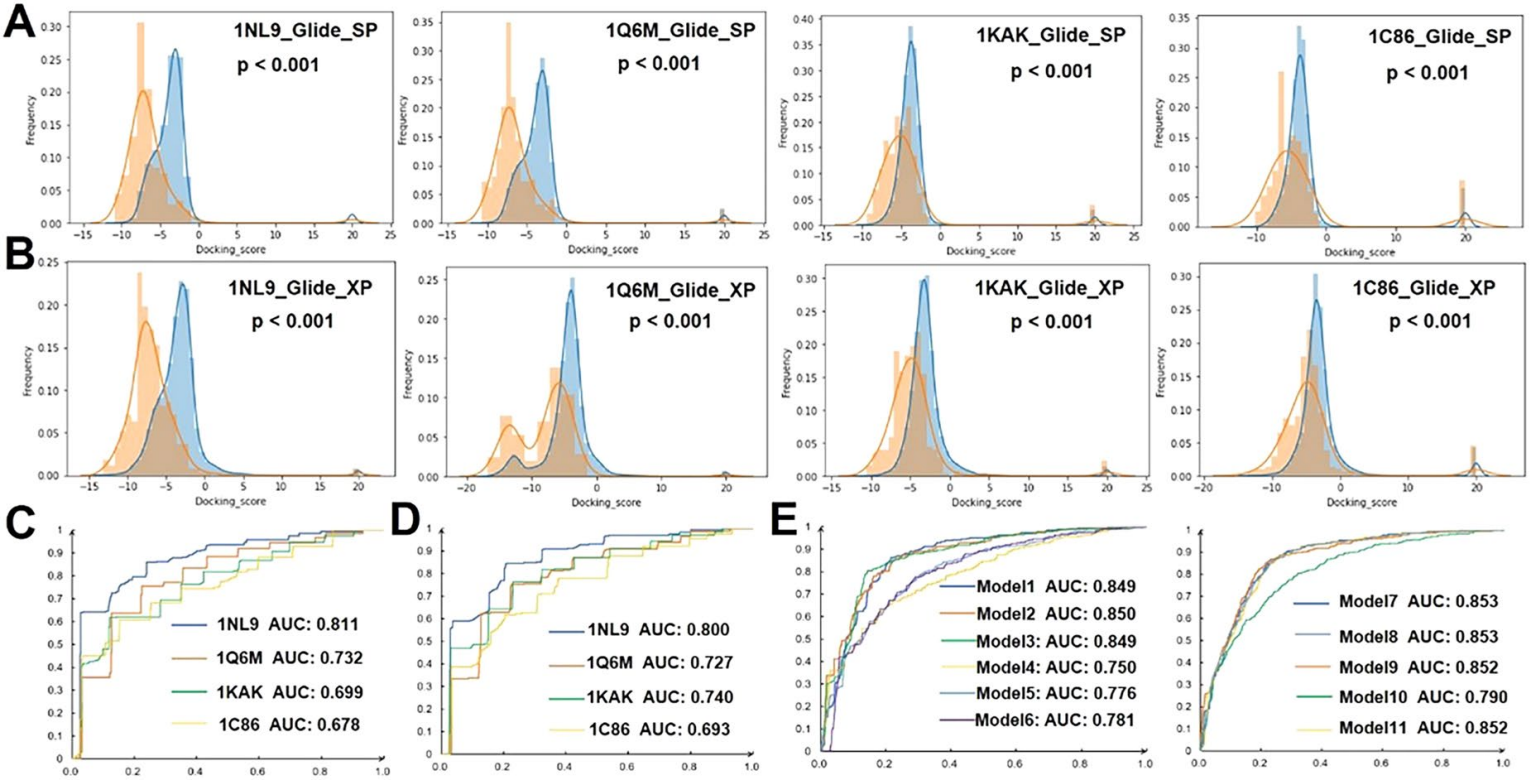
The docking accuracy of Glide_SP and Glide_XP with various crystal structures and multiple conformation models. A. Distributions of the docking scores for active inhibitors (orange) and decoys (blue) with different crystal structures docked by Glide_SP; B. Distributions of the docking scores for active inhibitors (orange) and decoys (blue) with different crystal structures docked by Glide_XP; C. ROC curves of NBC based on a single conformation docked by Glide_SP; D. ROC curves of NBC based on a single conformation docked by Glide_XP; E. ROC curves of NBC based on multiple conformations docked by Glide_SP (Model1: 1NL9 and 1Q6M, Model2: 1NL9 and 1KAK, Model3: 1NL9 and 1C86, Model4: 1C86 and 1KAK, Model5: 1C86 and 1Q6M; Model6: 1Q6M and 1KAK, Model7: 1NL9, 1Q6M, and 1KAKA, Model8: 1NL9, 1Q6M, and 1C86, Model9: 1NL9, 1KAK, and 1C86, Model10:1Q6M, 1KAK, and 1C86, Model11: 1NL9, 1Q6M, 1KAK, and 1C86).

Docking accuracy represented the ability of programs to discriminate active inhibitors from decoys. According to the results of docking reliability, the docking accuracy of 1C86, 1KAK, 1NL9, and 1Q6M by Glide_SP and Glide_XP were further evaluated by the Mann-Whitney U test. As shown in Figure 4(A) and (B), both Glide_SP and Glide_XP could effectively distinguish active inhibitors and decoys for all crystal structures with a very low *p* values.

The NBC models, in which the docking score was set as an independent property, were further constructed to intuitively estimate the discrimination capability of each crystal structure. As described in Figure 4(C), the area under curve (AUC) values of 1NL9 and 1Q6M docked by Glide_SP were 0.811 and 0.732, respectively, which was superior or comparable to Glide_XP (Figure 4D). For both docking program, the AUC values were higher than 0.7, which illustrated that these models displayed a satisfactory degree of distinction. Given there was no significant difference between Glide_SP and Glide_XP and the docking efficiency of Glide_SP was superior to Glide_XP, so Glide_SP was used for further research.

To improve the docking accuracy, the NBC models based on multiple crystal structures under Glide_SP were developed. Figure 4(E) showed that the AUC values increased after the combination of multiple structures, especially for the models containing 1NL9; the AUC values for these models remained around 0.85, which was preferable for single structure models.

### Virtual screening

**Figure 5.**
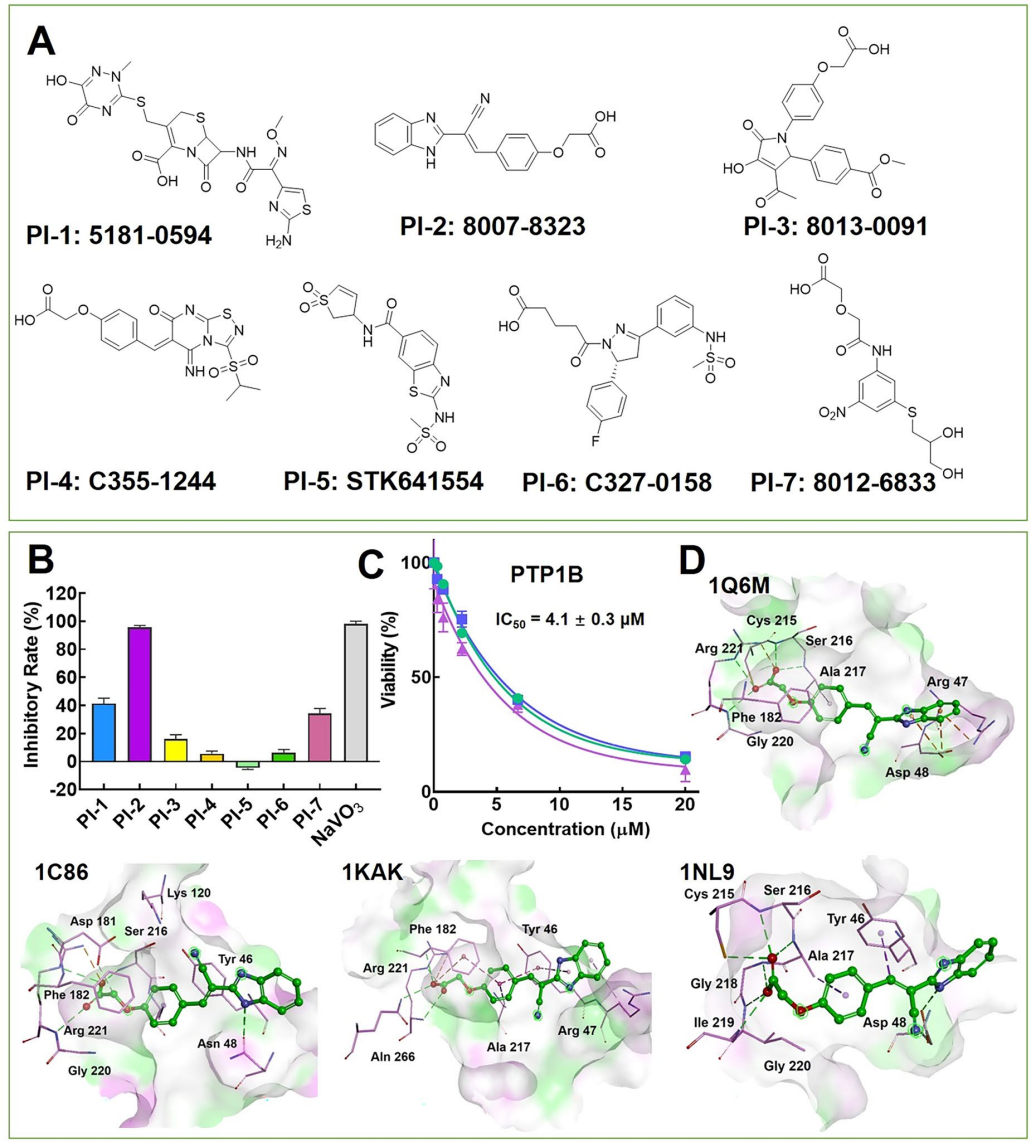
The structures, activities and binding modes of selected compounds. A. The structures of selected compounds through virtual screening; B. PTP1B inhibitory rate of selected compounds at the concentration of 20 μM; C. The IC_50_ value of PI-2 against PTP1B; D. The binding modes of PI-2 with PTP1B through molecular docking.

Eventually, a virtual screening pipeline consisted of pharmacophore screening based on 1NWL and molecular docking based on multi-conformational NBC model was conducted. After pharmacophore screening, around 1% compounds were kept for further screening, and top 100 compounds were remained for manual selection after molecular docking. To guarantee the diversity of the selected compounds, the top 100 compounds were clustered by the fingerprint FCFP_6 into 10 cluster. Finally, 7 compounds (Figure 5A) in different clusters were selected and purchased. These compounds were found with structural diversity, and most of them possessed a carboxylic group, which was preferable for interacting with the polar PTP1B active binding pocket.

The result of inhibitory rate measurement showed that compounds PI-1, PI-2 and PI-7 displayed a moderate to potent inhibitory activity against PTP1B at 20 μM (Figure 5B). Among them, compound PI-2 was the most potent, the inhibitory rate was presented as 95.6% which was comparable with positive control NaVO_3_ (Figure 5B). Enzymatic assay quantitatively clarified PI-2 was a moderate PTP1B inhibitor with IC_50_ = 4.1 ± 0.3 μM (Figure 5C).

### PTP1B binding mode and selectivity of PI-2

The binding mode of PI-2 with PTP1B was further studied by molecular docking. As shown in Figure 5(D), the carboxylic group of PI-2 effectively bound with the key residues in the active pocket of PTP1B through hydrogen bonds. Further, the phenyl group of PI-2 could interact with Phe 182 by hydrophobic interaction, and the benzimidazole group formed a hydrogen bond with Asn48/Asp48 or interacted with Tyr 46, Arg 47 through hydrophobic interaction to stabilise the binding conformation in different PTP1B crystal structures.

**Figure 6.**
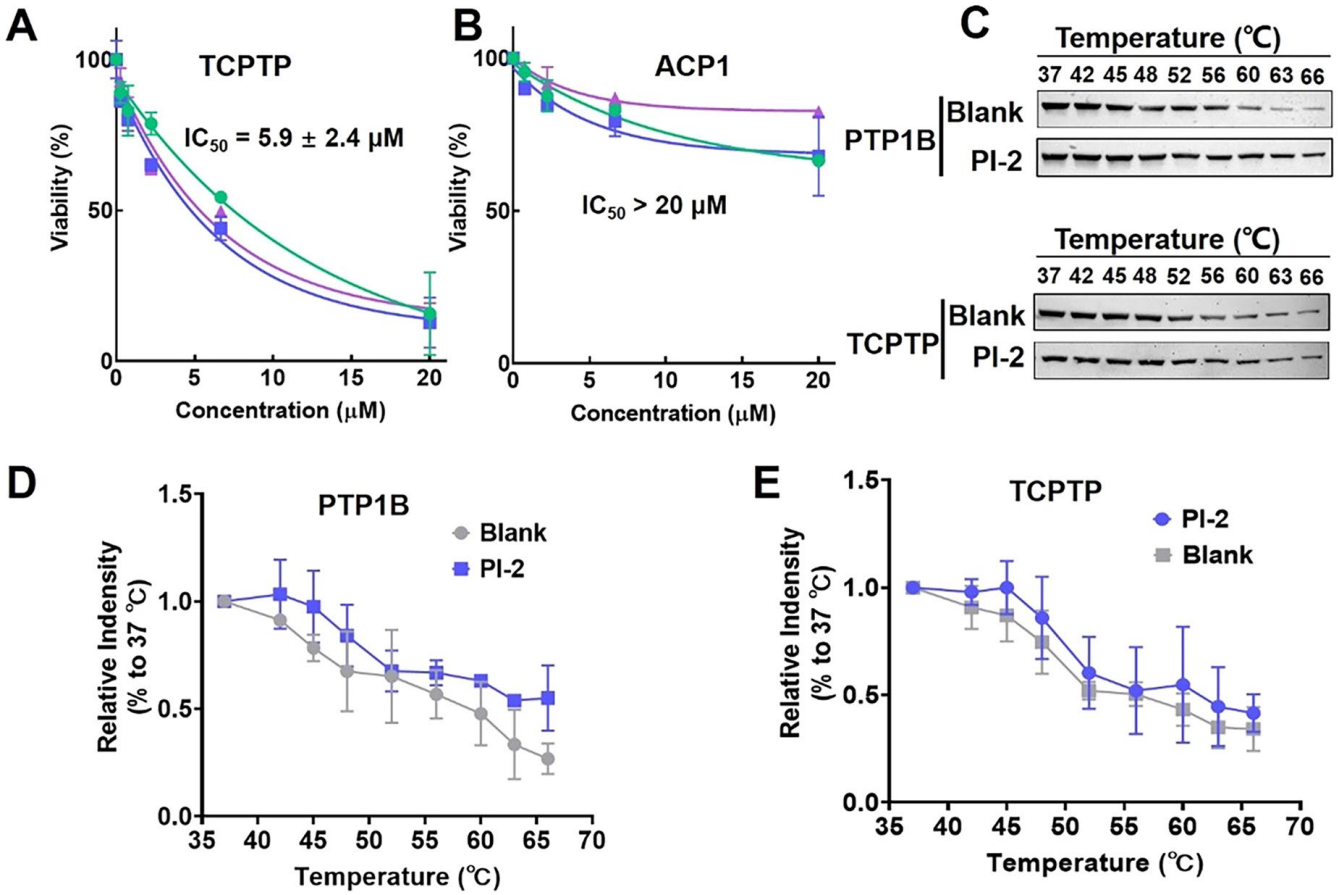
The PTP1B inhibitory selectivity of PI-2 at the enzymatic and the cellular level. A. The IC_50_ value of PI-2 against TCPTP; B. The IC_50_ value of PI-2 against ACP1; C-E. The effect of PI-2 on the stability of PTP1B and TCPTP in AC16 cells.

The inhibitory activity of PI-2 against TCPTP, the most homologous PTP with PTP1B, was measured to evaluate the PTP1B inhibitory selectivity of **PI-2**. As shown in Figure 6(A), the IC_50_ of PI-2 against TCPTP was 5.9 ± 2.4 μM, which was slightly higher than PTP1B with a selective index = 1.4 (selective index = IC_50 (TCPTP)_/IC_50 (PTP1B)_). As illustrated in the docking results, the secondary noncatalytic arylphosphate binding site consisted by Asn 48/Asp 48, Tyr 46, and Arg 47, which is not conserved for all PTPs, displayed an essential role for PI-2 binding. To further evaluate the PTP1B selectivity of PI-2, the inhibitory activity of PI-2 against acidic phosphatase (ACP1), which also contains this binding pocket, was measured. Compared with PTP1B, the inhibitory potency of PI-2 against ACP1 was much lower, with IC_50_ > 20 μM (Figure 6B), which means PI-2 exhibited a certain level of PTP1B selectivity at the enzymatic level.

To further clarify the PTP1B inhibitory activity and selectivity of PI-2 *in vitro*, the cell thermal shift assay (CETSA) was conducted. As illustrated in Figure 6(C)–(E), with the increase of temperature, the proteolysis level of PTP1B and TCPTP in the PI-2 treated group was much lower than that of the untreated group, demonstrating that PI-2 could effectively enhance the PTP1B and TCPTP stability *in vitro*. Further, for the PI-2 treated group, the PTP1B stability was higher than TCPTP, especially for high-temperature treatment, indicating PI-2 also displayed a moderate PTP1B inhibitory selectivity at the cellular level.

### The effect of PI-2 on LPS-induced AC16 apoptosis In vitro

**Figure 7.**
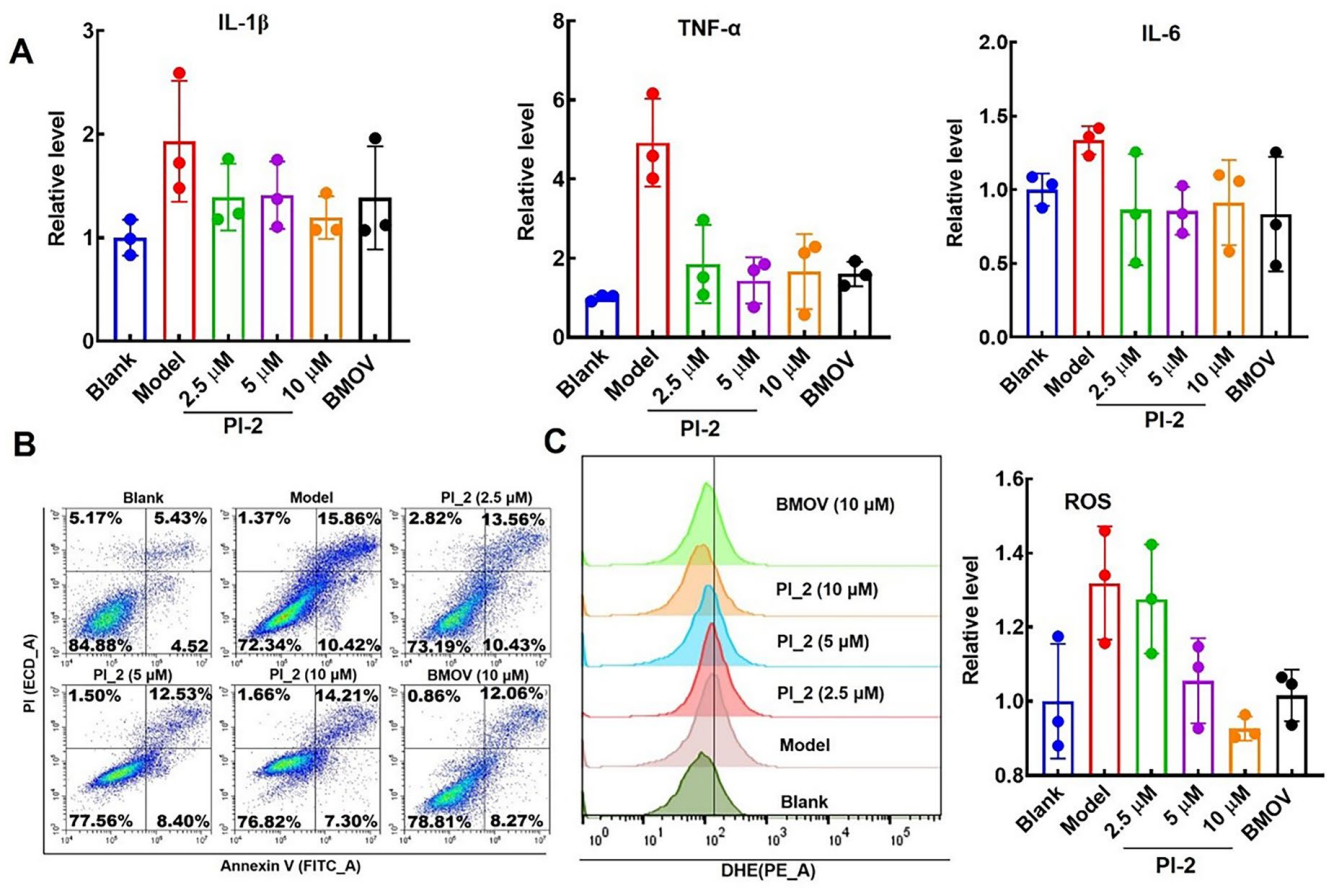
The effect of PI-2 on LPS-induced AC16 injury. A. The effect of PI-2 on LPS-induced AC16 inflammation; B. The effect of PI-2 on LPS-induced AC16 apoptosis; C. The effect of PI-2 on AC16 ROS level.

To investigate the effectiveness of PI-2 on the LPS-induced myocardial injury, the expressions of inflammation factors IL-1β, TNF-α, and IL-6 of AC16 cells were tested. We found that after LPS administration, the levels of IL-1β, TNF-α, and IL-6 were increased significantly (Figure 7A). After 48 h of PI-2 treatment at a concentration of 2.5 μM, the level of TNF-α decreased significantly, and the contents of IL-1β and IL-6 were also recovered (Figure 7A), which preliminarily proved that PI-2 could effectively alleviate LPS-induced myocardial injury. Then, we observed the influence of PI-2 on LPS-induced apoptosis of AC16 cells by flow cytometric analysis. As described in Figure 7(B), the percentage of early and late apoptotic cells was elevated by LPS administration, and PI-2 treatment could ameliorate this situation at a low concentration, which was comparable with 10 μM BMOV, the general PTP inhibitor.

The ROS level displayed a vital role in cell survival, so we further investigated the ROS scavenging activity of PI-2 in the AC16 cell. As expected, the level of ROS was significantly decreased after PI-2 treatment in a concentration-dependent manner (Figure 7C), suggesting PI-2 suppressed LPS-induced myocardial apoptosis through ROS scavenging.

### The mechanism of PI-2 on LPS-induced AC16 apoptosis

**Figure 8.**
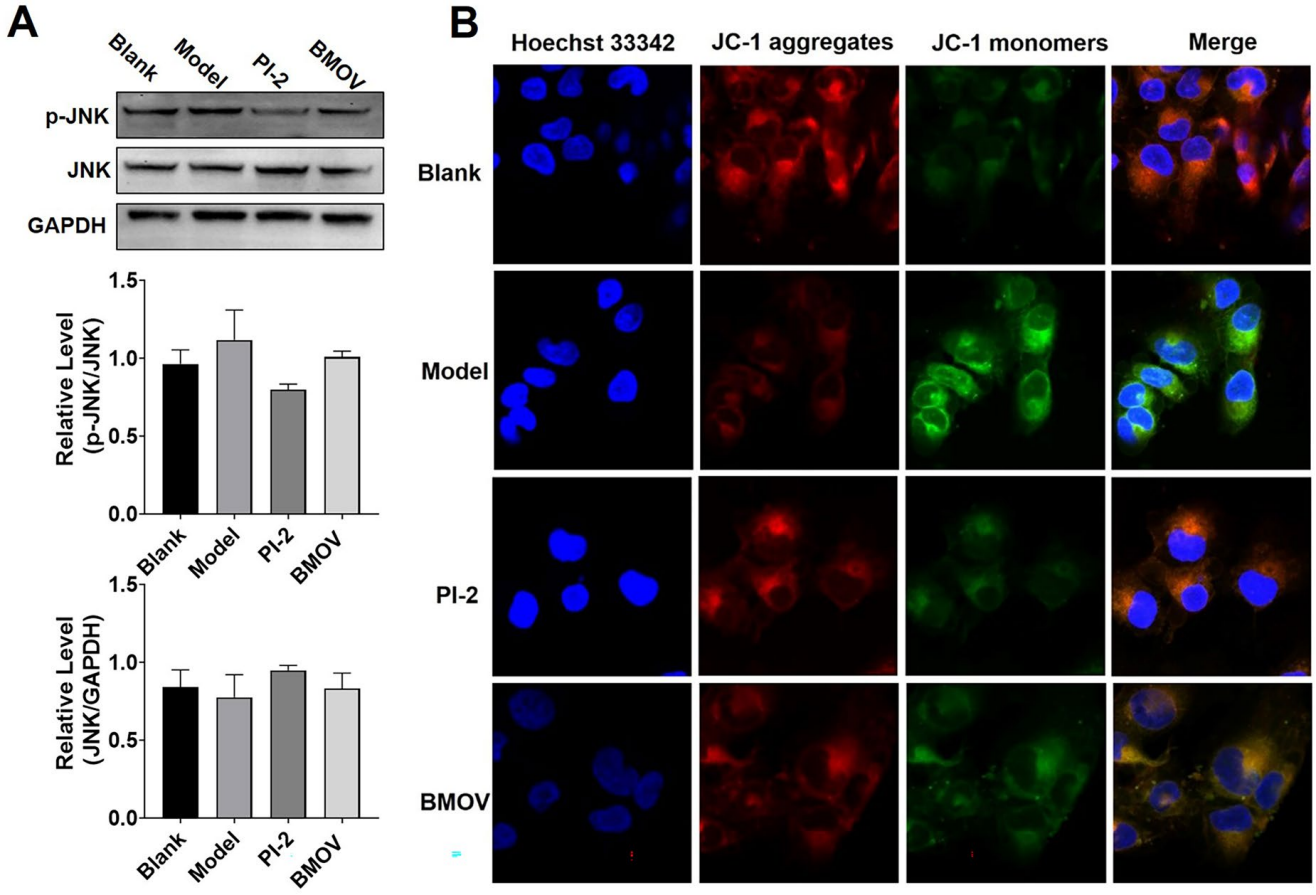
The effect of PI-2 on mitochondrial membrane potential through PTP1B/JNK pathway. A. The effect of PI-2 on the phosphorylation level of JNK; B. The effect of PI-2 on mitochondrial membrane potential.

Mitochondria are the main organelle to produce ROS and display a vital role in cell survival^41^. As JNK regulates the activity of mitochondrial apoptosis-related proteins directly^42^. Previous studies have demonstrated that inhibition of PTP1B attenuated activation of JNK^43^, so we speculated that PI-2 regulated LPS-induced AC16 apoptosis by maintaining mitochondrial potential through PTP1B/JNK pathway. To evaluate the function of PI-2 on AC16 mitochondria, first, we investigated the effect of PI-2 on the phosphorylation level of JNK. As shown in Figure 8(A), there is no obvious change for the expression of JNK after LPS and PI-2 treatment. However, the phosphorylation level of JNK was elevated by LPS and decreased after PI-2 treatment. These results further illustrated PI-2 was a preferable PTP1B inhibitor which could attenuate the activation of JNK. Then, the ΔΨm of mitochondrial membrane was tested by JC-1 staining (Figure 8B). In comparison with the Blank, LPS decreased the ΔΨm significantly. After the treatment of PI-2, the membrane potential of AC16 recovered, and a similar effect was seen with the treatment of BMOV. These results demonstrated that PI-2 was a potent PTP1B inhibitor that alleviated LPS-induced apoptosis by protecting mitochondrial membrane potential.

Besides, the study clarifies the anti-SICD activity of the novel inhibitor through PTP1B/JNK pathway. All the studies are carried out by LPS induced AC16 injury model, which could simulate the inflammatory factor releasing, oxidative stress processes induced by endotoxin release due to Gram-negative bacterial infection. However, the occurrence of SICD is more complicated, the model lacks real intercellular interactions and microenvironments *in vivo*, so further *in vivo* studies should be carried out and structure optimisation studies based on AI assisted or traditional drug design should be continued to find more potent PTP1B inhibitors.

## Conclusions

PTP1B is a potential target for sepsis-induced cardiovascular dysfunction. Many efforts have been made to discover potent and selective PTP1B inhibitors; However, the effect of these inhibitors on SICM is unclear.

In this study, we established a novel virtual screening pipeline with a multi-conformational NBC model. Compound PI-2, with IC_50_ = 4.2 μM was successfully screened by using this pipeline. Enzymatic and cellular assay illustrated that PI-2 displayed a moderate selectivity against both PTP1B and TCPTP, which was more than 4 times potent than ACP1. Biological assay demonstrated PI-2 was a potential compound for LPS-induced cardiovascular dysfunction treatment by improving mitochondrial membrane potential through inhibition of JNK phosphorylation. In summary, this study provides a potential PTP1B inhibitor and offers a new screening pipeline for discovering novel PTP1B inhibitors.

These findings serve as a pivotal stepping stone for the development of novel anti-SICD drugs. The anti-LPS induced cardiovascular dysfunction activity and substantial PTP1B inhibitory activity makes PI-2 as a potential candidate for disease involving PTP1B/JNK pathway. Future studies should delve into structure optimisation for bioactivity enhancement and nanomedicine and combination therapies should be considered to promote the clinical use of PI-2.”

## Materials and methods

### PTP1B crystal structure collection

After obtaining the distance matrix, the K-means algorithm (an unsupervised learning method) was applied to cluster the data and select representative PDB structures. To identify the optimal number of clusters (K), values ranging from 2 to 14 were evaluated. Clustering quality was assessed using the Silhouette Score, a metric that measures how well each data point fits within its assigned cluster compared to others. The score ranges from −1 to 1, with higher values indicating more well-defined and distinct clusters.

### PTP1B inhibitor dataset generation

PTP1B inhibitors were collected from ChEMBL (https://www.ebi.ac.uk/chembl/) database. Given that the IC_50_ was affected by assay condition, compounds with *K*_i_ value less than 10 μM were retained. The dataset was further refined by omitting allosteric inhibitors, and 501 inhibitors were kept. The decoys were generated by DUD-E (https://dude.docking.org/) with a ratio of 50 per 1 active compound. Finally, the database was divided into the training set and the test set with the ratio of 4:1, and the principal components of each set were analysed by Discovery Studio2019 (https://www.3ds.com/products-services/biovia/products/molecular-modeling-simulation/biovia-discovery-studio/).

### Pharmacophore generation and assessment

Pharmacophores were generated according to the receptor-ligand complexes by Discovery Studio2019. At most 10 pharmacophores were generated for each complex. The number of features in each pharmacophore was set as 4 to 6 and validated by the training set. The mode of conformation generation for each ligand in the training set was “FAST” and fitted to the pharmacophore “Flexible”.

The first pharmacophore generated by each complex was further validated by the test set. After pharmacophore fitting, the FitValue of each ligand was recorded. For those ligands that failed to fit in the pharmacophore, the FitValue was set as −10. Collecting all the ligands and the property of “Real_activity” was added for all ligands. The value of “1” was defined to active PTP1B inhibitors, and “0” was set for decoys. 5 folds cross-validation was used to generate Bayesian Model according to the “FitValue” and “Real_activity” and the AUC was calculated for each pharmacophore.

### General docking procedure

The docking prediction ability of four docking programs, including CDOCKER (Discovery Studio2019), Glide, Autodock (version 4.2.6, http://autodock.scripps.edu), and Autodock_Vina (version 1.1.2, https://vina.scripps.edu/) was assessed. The docking set was determined by the receptor-ligand complex. All the docking parameters for each program were set to default values unless otherwise stated.

CDOCKER: Proteins were prepared by the “Prepare Protein” module in Discovery Studio2019 with the default values, and ligands were prepared by the “Prepare Ligands” module with the pH range from 7.3 to 7.5. Molecular docking was conducted with the default parameters.

Glide: Proteins were prepared by the “Protein Preparation Wizard” module with the OPLS3 force field. Ligands were prepared by the “LigPrep” with pH = 7.4 ± 0.1. The “Glide Generation” module was used to generate grid files of each protein. Two docking modes, standard precision (SP) and extra precision (XP), were employed without any constraint.

Autodock: AutoDock_Tools_1.5.7 was used for protein and ligand preparation, including deleting water, adding hydrogen, assigning Gasteiger charges, and setting torsion count for ligands. The numbers of grid points in the xyz-direction were set according to the ligand in the crystal, and the grid spacing was 0.375 angstrom. The Lamarckian genetic algorithm (LGA) was used for conformation calculation.

Autodock_vina: The procedure was the same with Autodock, except the size of the search volume box was set as 20 Å × 20 Å × 20 Å.

### Assessment of docking predictions

The docking reliability and accuracy of these docking programs for each crystal structure were assessed. For docking reliability, the native ligand in the complex was extracted and re-docked into the defined docking site. Then, the RMSD of the docked poses with the native conformation of each ligand was calculated by Discovery Studio 2019. The top-scored pose with the highest docking score and the best pose that had the lowest RMSD with the native conformation were evaluated.

For docking accuracy, all the ligands in both the training set and the test set were docked into each protein with the Glide programs. The Mann-Whitney U test was used to evaluate the difference in the distribution of docking scores between active inhibitors and decoys. The receiver operating characteristic (ROC) curve and area under the curve (AUC) of each protein were generated and calculated through the NBC model. For this model, the docking score of each ligand was set as an independent property, and the “Real_activity” was set as a dependent property.

### Virtual screening

The final virtual screening pipeline consisted of Pharmacophore filtering produced by 1NWL, and Glide docking_SP with 1KAK, 1C86, 1NL9, and 1Q6M. After docking, the top 100 ligands were kept. Finally, the compounds were selected by comprehensively considering the FitValue and docking_score.

### Assessment of PTP1B inhibitory activity

Inhibitory rate: Phosphatase inhibitory activities were performed in the buffer solution containing 50 mM Hepes and 1 mM DTT at pH of 7.0. All the compounds (10 μM as the final concentration) were incubated with the tested enzyme for 10 min at 37 °C (solution without test compounds was set as blank control). Para-nitrophenyl phosphate (pNPP) was added (the final concentration was 5 mM) and incubated. Finally, the reaction was stopped by adding 3 M NaOH solution. The absorbance was measured at 405 nm, and the inhibitory rate was calculated.

IC_50_: The procedure was the same as the inhibitory rate assay except the concentrations were set as 10 μM, 5 μM, 2.5 μM, 1.25 μM, and 0.62 μM.

### Cell culture

AC16 human cardiomyocytes (Procell, Cat. NO.: CL-0790, CVCL_4U18) were cultured in Dulbecco’s modified Eagle’s medium (DMEM) supplemented with 10% foetal bovine serum (Procell, Cat. NO.: 164220), 100 units/mL penicillin, and 100 μg/mL streptomycin at 37 °C with 5% CO_2_ atmosphere incubator.

### Cell apoptosis assay

Cell apoptosis was detected by the Annexin V-FITC/PI apoptosis detection kit. After 24 h of treatment by 50 μg/mL LPS and tested compounds, cells were collected and washed with PBS. Cells were re-suspended in 100 μL Binding Buffer. Then, 5 μL Annexin V-FITC and 10 μL PI staining solution were added and reacted for 15 min. Finally, the results were measured by BD FACSCelesta flow cytometry (New York, USA). The data was analysed by FlowJo_V10.

### Reactive oxygen species (ROS) assay

The intracellular ROS was assessed using a ROS assay kit. After 24 h of treatment, cells were washed with PBS and stained with dichlorodihydrofluores-ceindiacetate (DCFH-DA) at 37 °C for 30 min in the dark. Then, samples were detected by FACSCelesta flow cytometry.

### Mitochondrial membrane potential assay

The mitochondrial membrane potential was measured with JC-1 MMP assay kit. Cells were treated with the tested compounds for 24 h. and stained with JC-1 at 37 °C for 20 min. Then, the cells were washed with JC-1 staining buffer for twice. Finally, cell culture medium was added and evaluated through fluorescence microscope.

### Western blot analysis

The primary antibody PTP1B (Rabbit, dilution: 1: 2000, RRID number: AB_10642566, Cat. NO.: 11334–1-AP) and TCPTP (Rabbit, dilution: 1: 500, RRID number: AB_2173235, Cat. NO.: 11214–1-AP) were purchased from Proteintech. JNK (Rabbit, dilution: 1:2000, Cat. NO.: YM8450) and p-JNK (Rabbit, dilution: 1:2000, Cat. NO.: YP0157) were purchased from Immunoway. The secondary antibody was purchased from Invitrogen (Goat, dilution: 1: 5000, RRID number: AB_1185567, Cat. NO.: Cat. NO.: 32460). The regular range protein markers were purchased from Yeasen (Cat. NO.: 20351ES72) for PTP1B and TCPTP and Thermo (Cat. NO.: 26616) for JNK and p-JNK.

After 24 h of treatment, proteins were extracted by RIPA cell lysis buffer (Beyotime, P0013B) with protease (MCE, HY-K0010) and phosphatase inhibitor cocktail (MCE, HY-K0010). After concentration determination by BCA assay, equal amounts of proteins were loaded for SDS-PAGE and transferred onto PVDF membrane. The membrane was further blocked with commercial blocking solution (Beyotime, China) and incubated with primary antibodies and secondary antibodies subsequently. Bands were visualised by ECL chemiluminescence kit (MCE, HY-K1005) and quantified by ImageJ software.

### Statistical analysis

Statistical analysis was performed by GraphPad Prism 8 (GraphPad Software, Inc, La Jolla, CA, USA). All statistics are shown as mean ± SD with *n* = 3 unless otherwise specified. Analysis of variance (ANOVA) was used for comparisons of multiple groups. *P* < 0.05 was considered significant differences.

## Data Availability

Data will be made available on request.

## References

[1] De Backer D, Deutschman CS, Hellman J, Myatra SN, Ostermann M, Prescott HC, Talmor D, Antonelli M, Pontes Azevedo LC, Bauer SR, Surviving Sepsis Campaign Research Committee, et al. Surviving sepsis campaign research priorities 2023. Crit Care Med. 2024;52(2):268–296. 38240508 10.1097/CCM.0000000000006135

[2] Schlapbach LJ, Watson RS, Sorce LR, Argent AC, Menon K, Hall MW, Akech S, Albers DJ, Alpern ER, Balamuth F, Society of Critical Care Medicine Pediatric Sepsis Definition Task Force, et al. International consensus criteria for pediatric sepsis and septic shock. JAMA. 2024;331(8):665–674. 38245889 10.1001/jama.2024.0179 PMC10900966

[3] Liu H, Xu C, Hu Q, Wang Y. Sepsis-induced cardiomyopathy: understanding pathophysiology and clinical implications. Arch Toxicol. 2025;99(2):467–480. 39601874 10.1007/s00204-024-03916-x

[4] Yu X, Gao J, Zhang C. Sepsis-induced cardiac dysfunction: mitochondria and energy metabolism. Intensive Care Med Exp. 2025;13(1):20. 39966268 10.1186/s40635-025-00728-w PMC11836259

[5] Habimana R, Choi I, Cho HJ, Kim D, Lee K, Jeong I. Sepsis-induced cardiac dysfunction: a review of pathophysiology. Acute Crit Care. 2020;35(2):57–66. 32506871 10.4266/acc.2020.00248 PMC7280799

[6] Gao L, Shi Q, Sun B, Zhang X, Zheng P, Zhou L, Tian G, Li H. c-FLIP protects cardiac microcirculation in sepsis-induced myocardial dysfunction via FUNDC1-mediated regulation of mitochondrial autophagy. JACC Basic Transl Sci. 2025;10(8):101257. 40372306 10.1016/j.jacbts.2025.02.016 PMC12399170

[7] She H, Tan L, Du Y, Zhou Y, Guo N, Zhang J, Du Y, Wang Y, Wu Z, Ma C, et al. VDAC2 malonylation participates in sepsis-induced myocardial dysfunction via mitochondrial-related ferroptosis. Int J Biol Sci. 2023;19(10):3143–3158. 14 37416771 10.7150/ijbs.84613 PMC10321281

[8] Xu Y, Zhang S, Rong J, Lin Y, Du L, Wang Y, Zhang Z. Sirt3 is a novel target to treat sepsis induced myocardial dysfunction by acetylated modulation of critical enzymes within cardiac tricarboxylic acid cycle. Pharmacol Res. 2020;159:104887. 32526680 10.1016/j.phrs.2020.104887

[9] Chen D, Hou Y, Cai X. MiR-210-3p enhances cardiomyocyte apoptosis and mitochondrial dysfunction by targeting the NDUFA4 gene in sepsis-induced myocardial dysfunction. Int Heart J. 2021;62(3):636–646. 33994501 10.1536/ihj.20-512

[10] Agrawal N, Dhakrey P, Pathak S. A comprehensive review on the research progress of PTP1B inhibitors as antidiabetics. Chem Biol Drug Des. 2023;102(4):921–938. 37232059 10.1111/cbdd.14275

[11] Coronell-Tovar A, Pardo JP, Rodríguez-Romero A, Sosa-Peinado A, Vásquez-Bochm L, Cano-Sánchez P, Álvarez-Añorve LI, González-Andrade M. Protein tyrosine phosphatase 1B (PTP1B) function, structure, and inhibition strategies to develop antidiabetic drugs. FEBS Lett. 2024;598(15):1811–1838. 38724486 10.1002/1873-3468.14901

[12] Feng B, Dong YQ, Shang B, et al. Convergent protein phosphatase inhibitor design for PTP1B and TCPTP: Exchangeable vanadium coordination complexes on Graphene Quantum Dots. Advanc Funct Mater. 2022;32:2108645.

[13] Bence KK, Delibegovic M, Xue B, Gorgun CZ, Hotamisligil GS, Neel BG, Kahn BB. Neuronal PTP1B regulates body weight, adiposity and leptin action. Nat Med. 2006;12(8):917–924. 16845389 10.1038/nm1435

[14] Patel AD, Pasha TY, Lunagariya P, Shah U, Bhambharoliya T, Tripathi RKP. A library of thiazolidin-4-one derivatives as protein tyrosine phosphatase 1B (PTP1B) inhibitors: An attempt to discover novel antidiabetic agents. ChemMedChem. 2020;15(13):1229–1242. 32390300 10.1002/cmdc.202000055

[15] Balaramnavar VM, Srivastava R, Rahuja N, Gupta S, Rawat AK, Varshney S, Chandasana H, Chhonker YS, Doharey PK, Kumar S, et al. Identification of novel PTP1B inhibitors by pharmacophore based virtual screening, scaffold hopping and docking. Eur J Med Chem. 2014;87:578–594. 25299681 10.1016/j.ejmech.2014.09.097

[16] Liu C, Xiang J, Chen Y, He C, Tong J, Liao Y, Lei H, Sun L, Yao G, Xie Z, et al. MiR-125a-5p in MSC-derived small extracellular vesicles alleviates Müller cells injury in diabetic retinopathy by modulating mitophagy via PTP1B pathway. Cell Death Discov. 2025;11(1):226. 40341376 10.1038/s41420-025-02439-3 PMC12062395

[17] Shum M, Shintre CA, Althoff T, Gutierrez V, Segawa M, Saxberg AD, Martinez M, Adamson R, Young MR, Faust B, et al. ABCB10 exports mitochondrial biliverdin, driving metabolic maladaptation in obesity. Sci Transl Med. 2021;13(594):eabd1869. 34011630 10.1126/scitranslmed.abd1869 PMC8300486

[18] Hu C, Li G, Mu Y, Wu W, Cao B, Wang Z, Yu H, Guan P, Han L, Li L, et al. Discovery of anti-TNBC agents targeting PTP1B: Total synthesis, structure-activity relationship, in vitro and in vivo investigations of Jamunones. J Med Chem. 2021;64(9):6008–6020. 33860662 10.1021/acs.jmedchem.1c00085

[19] Fu F, Liu C, Shi R, Li M, Zhang M, Du Y, Wang Q, Li J, Wang G, Pei J, et al. Punicalagin protects against diabetic cardiomyopathy by promoting Opa1-mediated mitochondrial fusion via regulating PTP1B-Stat3 pathway. Antioxid Redox Signal. 2021;35(8):618–641. 33906428 10.1089/ars.2020.8248

[20] Liu F, Chen J, Hu W, Gao C, Zeng Z, Cheng S, Yu K, Qian Y, Xu D, Zhu G, et al. PTP1B inhibition improves mitochondrial dynamics to alleviate calcific aortic valve disease via regulating OPA1 homeostasis. JACC Basic Transl Sci. 2022;7(7):697–712. 35958694 10.1016/j.jacbts.2022.03.002 PMC9357565

[21] Xie W-J, Liu M, Zhang X, Zhang Y-G, Jian Z-H, Xiong X-X. Astaxanthin suppresses LPS-induced myocardial apoptosis by regulating PTP1B/JNK pathway in vitro. Int Immunopharmacol. 2024;127:111395. 38141411 10.1016/j.intimp.2023.111395

[22] Coquerel D, Neviere R, Delile E, Mulder P, Marechal X, Montaigne D, Renet S, Remy-Jouet I, Gomez E, Henry J-P, et al. Gene deletion of protein tyrosine phosphatase 1B protects against sepsis-induced cardiovascular dysfunction and mortality. Arterioscler Thromb Vasc Biol. 2014;34(5):1032–1044. 24578383 10.1161/ATVBAHA.114.303450

[23] Delile E, Nevière R, Thiébaut P-A, Maupoint J, Mulder P, Coquerel D, Renet S, Rieusset J, Richard V, Tamion F, et al. Reduced insulin resistance contributes to the beneficial effect of protein tyrosine phosphatase-1B deletion in a mouse model of sepsis. Shock. 2017;48(3):355–363. 28272165 10.1097/SHK.0000000000000853

[24] Yang L, Chen F, Gao C, Chen J, Li J, Liu S, Zhang Y, Wang Z, Qian S. Design and synthesis of tricyclic terpenoid derivatives as novel PTP1B inhibitors with improved pharmacological property and in vivo antihyperglycaemic efficacy. J Enzyme Inhib Med Chem. 2020;35(1):152–164. 31742469 10.1080/14756366.2019.1690481 PMC6882489

[25] Bongard RD, Lepley M, Thakur K, Talipov MR, Nayak J, Lipinski RAJ, Bohl C, Sweeney N, Ramchandran R, Rathore R, et al. Serendipitous discovery of light-induced (In Situ) formation of an Azo-bridged dimeric sulfonated naphthol as a potent PTP1B inhibitor. BMC Biochem. 2017;18(1):10. 28569147 10.1186/s12858-017-0083-3 PMC5452347

[26] Zhang S, Zhang ZY. PTP1B as a drug target: recent developments in PTP1B inhibitor discovery. Drug Discov Today. 2007;12(9–10):373–381. 17467573 10.1016/j.drudis.2007.03.011

[27] Debnath A, Rani A, Mazumder R, et al. Discovery of novel PTP1B inhibitors by high-throughput virtual screening. Curr Comput Aided Drug Des. 2026;22(1):61–86. 10.2174/0115734099278007241004105500 39411941

[28] Liang S, Tran E, Du X, Dong J, Sudholz H, Chen H, Qu Z, Huntington ND, Babon JJ, Kershaw NJ, et al. A small molecule inhibitor of PTP1B and PTPN2 enhances T cell anti-tumor immunity. Nat Commun. 2023;14(1):4524. 37500611 10.1038/s41467-023-40170-8 PMC10374545

[29] Huang Q, Hu L, Chen H, Yang B, Sun X, Wang M. A medicinal chemistry perspective on protein tyrosine phosphatase nonreceptor type 2 in tumor immunology. J Med Chem. 2025;68(4):3995–4021. 39936476 10.1021/acs.jmedchem.4c01802

[30] Khator R, Biharee A, Bhatia N, Kulkarni S, Singh Y, Karthikeyan C, Jain AK, Thareja S. Medicinal aspects of PTP1B inhibitors as anti-breast cancer agents: An overview. Curr Med Chem. 2024;31(34):5535–5549. 37711015 10.2174/0929867331666230914103645

[31] Jia Z, Ye Q, Dinaut AN, Wang Q, Waddleton D, Payette P, Ramachandran C, Kennedy B, Hum G, Taylor SD, et al. Structure of protein tyrosine phosphatase 1B in complex with inhibitors bearing two phosphotyrosine mimetics. J Med Chem. 2001;44(26):4584–4594. 11741477 10.1021/jm010266w

[32] Wan Z-K, Follows B, Kirincich S, Wilson D, Binnun E, Xu W, Joseph-McCarthy D, Wu J, Smith M, Zhang Y-L, et al. Probing acid replacements of thiophene PTP1B inhibitors. Bioorg Med Chem Lett. 2007;17(10):2913–2920. 17336064 10.1016/j.bmcl.2007.02.043

[33] Xin Z, Oost TK, Abad-Zapatero C, Hajduk PJ, Pei Z, Szczepankiewicz BG, Hutchins CW, Ballaron SJ, Stashko MA, Lubben T, et al. Potent, selective inhibitors of protein tyrosine phosphatase 1B. Bioorg Med Chem Lett. 2003;13(11):1887–1890. 12749891 10.1016/s0960-894x(03)00302-0

[34] Klopfenstein SR, Evdokimov AG, Colson A-O, Fairweather NT, Neuman JJ, Maier MB, Gray JL, Gerwe GS, Stake GE, Howard BW, et al. 1,2,3,4-Tetrahydroisoquinolinyl sulfamic acids as phosphatase PTP1B inhibitors. Bioorg Med Chem Lett. 2006;16(6):1574–1578. 16386905 10.1016/j.bmcl.2005.12.051

[35] Ala PJ, Gonneville L, Hillman M, Becker-Pasha M, Yue EW, Douty B, Wayland B, Polam P, Crawley ML, McLaughlin E, et al. Structural insights into the design of nonpeptidic isothiazolidinone-containing inhibitors of protein-tyrosine phosphatase 1B. J Biol Chem. 2006;281(49):38013–38021. 17028182 10.1074/jbc.M607913200

[36] Salmeen A, Andersen JN, Myers MP, Tonks NK, Barford D. Molecular basis for the dephosphorylation of the activation segment of the insulin receptor by protein tyrosine phosphatase 1B. Mol Cell. 2000;6(6):1401–1412. 11163213 10.1016/s1097-2765(00)00137-4

[37] Iversen LF, Andersen HS, Branner S, Mortensen SB, Peters GH, Norris K, Olsen OH, Jeppesen CB, Lundt BF, Ripka W, et al. Structure-based design of a low molecular weight, nonphosphorus, nonpeptide, and highly selective inhibitor of protein-tyrosine phosphatase 1B. J Biol Chem. 2000;275(14):10300–10307. 10744717 10.1074/jbc.275.14.10300

[38] Scapin G, Patel SB, Becker JW, Wang Q, Desponts C, Waddleton D, Skorey K, Cromlish W, Bayly C, Therien M, et al. The structural basis for the selectivity of benzotriazole inhibitors of PTP1B. Biochemistry. 2003;42(39):11451–11459. 14516196 10.1021/bi035098j

[39] Erlanson DA, McDowell RS, He MM, Randal M, Simmons RL, Kung J, Waight A, Hansen SK. Discovery of a new phosphotyrosine mimetic for PTP1B using breakaway tethering. J Am Chem Soc. 2003;125(19):5602–5603. 12733877 10.1021/ja034440c

[40] Szczepankiewicz BG, Liu G, Hajduk PJ, Abad-Zapatero C, Pei Z, Xin Z, Lubben TH, Trevillyan JM, Stashko MA, Ballaron SJ, et al. Discovery of a potent, selective protein tyrosine phosphatase 1B inhibitor using a linked-fragment strategy. J Am Chem Soc. 2003;125(14):4087–4096. 12670229 10.1021/ja0296733

[41] Xu X, Pang Y, Fan X. Mitochondria in oxidative stress, inflammation and aging: from mechanisms to therapeutic advances. Signal Transduct Target Ther. 2025;10(1):190. 40500258 10.1038/s41392-025-02253-4 PMC12159213

[42] Chen W-F, Tsai S-C, Zhang Y-H, Chang H-M, Wu W-J, Su J-H, Wu B-N, Chen C-Y, Lin M-Y, Chen H-L, et al. Rhopaloic acid A triggers mitochondria damage-induced apoptosis in oral cancer by JNK/BNIP3/Nix-mediated mitophagy. Phytomedicine. 2024;132:155855. 39043083 10.1016/j.phymed.2024.155855

[43] Zhao Y, Liu Y, Deng J, Zhu C, Ma X, Jiang M, Fan D. Ginsenoside F4 alleviates skeletal muscle insulin resistance by regulating PTP1B in type II diabetes mellitus. J Agric Food Chem. 2023;71(39):14263–14275. 37726223 10.1021/acs.jafc.3c01262

